# Co-expression networks identify distinct immune infiltrates in hepatocellular carcinoma

**DOI:** 10.1186/2051-1426-3-S2-P213

**Published:** 2015-11-04

**Authors:** Derek Y Chiang, Alan Huang

**Affiliations:** 1 Novartis Institutes for Biomedical Research, Cambridge, MA, USA

## Background

The vast majority of hepatocellular carcinoma (HCC) arise in the context of chronic inflammation, especially with hepatitis B or hepatitis C viral infection. Several studies have identified prognostic immune biomarkers in HCC tumors and peritumoral regions. Recently, a Phase 1 trial of a PD-1 inhibitor has demonstrated efficacy in HCC. In order to characterize the diversity of immune microenvironments in HCC, we investigated co-expression networks of immune lineage-specific genes.

## Results

We conducted a meta-analysis of gene expression data from over 500 HCC tumors and matched adjacent liver specimens. PD-L1 and PD-L2 had higher RNA levels in adjacent liver, compared with tumors. Tumoral expression of PD-L1 and PD-L2 were correlated with macrophage lineage genes. We identified 3 major co-expression network modules that corresponded with different immune cell sub-types: (1) An infiltrating T cell module was enriched for TCR activation, recruitment chemokines and elevated immune checkpoints. (2) A hepatic stellate cell module was associated with extracellular matrix remodeling, epithelial-to-mesenchymal transition and TGF-beta signaling. (3) A macrophage module had elevated macrophage lineage genes. By integrating these co-expression modules with HCC molecular sub-classes, the infiltrating T cell signature was enriched in the Hoshida S1 subclass^1^ and Chiang interferon subclass^2^, and was less prevalent among HCC with *CTNNB1* mutations.

## Conclusion

Transcriptomic analyses revealed immune cell types and potential regulators in HCC. The joint profiling of infiltrating immune sub-types and genetic alterations may guide the selection of combination therapies.
